# HALP score and 30-day mortality: an observational analysis of clinical associations in Pneumonia

**DOI:** 10.7717/peerj.21442

**Published:** 2026-06-11

**Authors:** Remzi Çetinkaya, Mehmet Özel, Ali Cankut Tatlıparmak, Songül Doğan Araç

**Affiliations:** 1 Department of Emergency Medicine, University of Health Sciences, Diyarbakır Gazi Yasargil Training and Research Hospital, Diyarbakir, Turkey; 2 Department of Emergency Medicine, Üsküdar University, İstanbul, Turkey

**Keywords:** Community-acquired pneumonia, HALP score, Mortality, Causal investigation

## Abstract

**Background:**

Community-acquired pneumonia (CAP) is a major cause of morbidity and mortality worldwide. Given the limitations of traditional risk scoring systems, new biomarkers such as the HALP (hemoglobin, albumin, lymphocyte, and platelet) score have emerged. This study evaluated the prognostic value of the HALP score in predicting 30-day mortality in patients with CAP and explored possible mechanisms underlying this association.

**Methods:**

A retrospective single-center study was conducted, including 467 adult patients hospitalized with pneumonia. HALP, PSI (pneumonia severity index), CURB-65 (confusion, uremia, respiratory rate, blood pressure, age ≥ 65 years), and NEWS (national early warning score) scores were calculated. ROC analysis was used to assess the prognostic value of HALP, and causal mediation analysis was performed to investigate the factors influencing the HALP–mortality relationship.

**Results:**

The 30-day mortality rate was 21.4%. Patients who died were older, mostly male, and had worse clinical and laboratory parameters, including lower HALP scores. The HALP score demonstrated good predictive ability (area under the receiver operating characteristic curve 0.794). Mediation analysis revealed that PSI, CURB-65, NEWS scores, confusion, oxygen saturation, shock index, and radiological findings significantly mediated the association between HALP and mortality.

**Conclusion:**

The HALP score was associated with short-term mortality in patients with CAP. Lower HALP values were associated with greater clinical severity and higher mortality, suggesting that it may serve as a complementary tool alongside traditional risk scoring systems.

## Introduction

Community-acquired pneumonia (CAP), when considered alongside other lower respiratory tract infections, ranks as the fourth leading cause of death worldwide (GBD 2016 Causes of Death Collaborators, 2017). In general, the 30-day mortality rate among patients hospitalized with pneumonia has been reported to range between 5% and 15% (Viasus et al., 2023). However, this rate can vary significantly depending on the patient’s clinical condition; for instance, it may exceed 30% in cases of severe pneumonia requiring intensive care (Chen et al., 2020). Early identification of severe CAP cases enables modifications in treatment strategies, thereby improving patient outcomes (Frenzen et al., 2018).

Various mortality prediction tools have been developed to determine the appropriate treatment setting and level of care for patients with CAP (Kwok et al., 2013). Among these tools, the Pneumonia Severity Index (PSI) and CURB-65 are the most widely recognized, validated, and frequently used scoring systems in clinical practice (Fine et al., 1997; Lim et al., 2003). While the Pneumonia Severity Index (PSI) is complex due to the inclusion of numerous variables, CURB-65 is more practical but may demonstrate lower sensitivity, particularly in younger patients (American Thoracic Society & Infectious Diseases Society of America, 2005). More general tools have also been developed to predict clinical deterioration regardless of etiology; among these, the National Early Warning Score (NEWS) is commonly used. Although effective in forecasting adverse clinical outcomes within the hospital setting, NEWS is not specific to pneumonia (Das et al., 2024). For these reasons, there has been growing interest in alternative prognostic markers in CAP that are easier to apply, based on laboratory parameters, and reflective of systemic inflammation and nutritional status. One such emerging biomarker is the HALP score (Hemoglobin, Albumin, Lymphocyte, and Platelet count). Initially introduced as a prognostic indicator in solid tumors, HALP has since been investigated in cardiovascular diseases, sepsis, and chronic inflammatory conditions. By combining hematological and nutritional parameters, the HALP score provides insight into the body’s capacity to respond to infection (Zhai et al., 2021; Li et al., 2025). A high HALP score, reflecting a combination of elevated hemoglobin, albumin, and lymphocyte levels with a low platelet count, indicates a favorable immuno-nutritional status, whereas a low HALP score may suggest malnutrition and immune dysfunction (Çolak et al., 2023).

We hypothesized that in diseases associated with systemic inflammation, such as pneumonia, the HALP score may have significant predictive value for mortality. In this study, we aimed to evaluate the predictive power of the HALP score for 30-day mortality in patients hospitalized with CAP and to investigate the potential mediating mechanisms underlying this association using causal mediation analysis.

Although the HALP score has been investigated in several inflammatory and critical illness settings, its prognostic value in pneumonia remains insufficiently explored. In addition, the potential clinical pathways linking HALP with adverse outcomes in pneumonia have not been clearly described. Therefore, this study aimed to evaluate the association between the HALP score and 30-day mortality in patients hospitalized with pneumonia and to explore potential clinical pathways related to this association using mediation analysis.

## Materials & Methods

### Study design and patient selection

This retrospective observational study was conducted at Diyarbakır Gazi Yasargil Training and Research Hospital. A total of 467 patients admitted to the hospital (including both the general ward and intensive care unit) with a diagnosis of pneumonia, who presented to the emergency department and for whom 30-day mortality data were available, were included in the study. Patients were enrolled between January 1, 2022, and December 31, 2024, based on their initial presentation to the emergency department with a clinical diagnosis of pneumonia. The cohort consisted of adult patients diagnosed with community-acquired pneumonia based on compatible clinical findings together with radiological evidence of new pulmonary infiltrates. All eligible patients presenting during the study period were consecutively included to reduce the risk of selection bias. This study was based on retrospective observational data; therefore, the findings should be interpreted as associations rather than causal relationships.

### Ethical approval

The study was approved by the Clinical Research Ethics Committee of Diyarbakır Gazi Yasargil Training and Research Hospital (Decision No: 0019, Date: April 19, 2024). The research was conducted in accordance with the Declaration of Helsinki, and written informed consent was obtained from each patient or, when unavailable, from the next of kin. Patient confidentiality was maintained throughout the study, and only anonymized data were used for analysis purposes.

Inclusion criteria: (1) Age 18 years or older, (2) diagnosis of pneumonia confirmed by clinical, radiological, and/or laboratory findings, (3) hospitalization due to pneumonia, (4) complete data available without missing values. Exclusion criteria: (1) pregnancy, (2) receiving immunosuppressive therapy, (3) cases with incomplete or erroneous records that precluded analysis.

### Data collection

Demographic characteristics (age, sex), comorbidities (hypertension, diabetes mellitus, cardiovascular diseases, cerebrovascular diseases, dementia, malignancy, *etc.*), vital signs (blood pressure, heart rate, peripheral oxygen saturation (SpO_2_), temperature), laboratory parameters (lymphocyte count, albumin, lactate, urea, *etc.*), and prognostic scores at admission (PSI, CURB-65, NEWS) were retrospectively obtained from the hospital information management system.

The HALP score (Hemoglobin × Albumin × Lymphocyte/Platelet) and CALLY index (C-reactive protein (CRP) × age/lymphocyte × albumin) were calculated for each patient using standard formulas. Only patients with complete laboratory and clinical data required for score calculations were included in the final analysis. No statistical imputation was performed. A data dictionary describing all variables included in the dataset has been provided alongside the raw dataset to facilitate transparency and reproducibility. A multivariable logistic regression model was constructed including HALP score, PSI, CURB-65, NEWS, and age, as these represent established severity indices and key demographic factors. Comorbidities were not included in the primary model to avoid overfitting and multicollinearity, given their partial inclusion within the PSI score. The primary multivariable regression analysis was performed on the full cohort, while the train–test split was used only for internal validation of model performance.

### Analysis

All statistical analyses were performed using R version 4.4.0 (R Core Team, 2024). Baseline characteristics were compared between survivors and non-survivors using the Student’s *t*-test or Wilcoxon rank-sum test for continuous variables, and the chi-square test or Fisher’s exact test for categorical variables. Continuous variables were presented as mean ± standard deviation or median (IQR) based on distributional characteristics. The predictive performance of the HALP score was evaluated using receiver operating characteristic (ROC) analysis. The area under the ROC curve (AUROC) with 95% confidence intervals (CIs), Youden-optimal threshold, sensitivity, specificity, and likelihood ratios (+LR, –LR) were calculated. To explore potential pathways linking the HALP score with 30-day mortality, an exploratory mediation analysis was conducted using the mediation package in R. HALP score was modeled as the exposure, 30-day mortality as the outcome, and 35 clinical variables as candidate mediators. Candidate mediators were selected based on clinically relevant variables available at admission, including established pneumonia severity indices, vital signs, laboratory parameters, and major comorbidities. For each candidate, a linear regression model estimated the effect of HALP on the mediator, followed by a logistic regression model including both HALP and the mediator to predict mortality. Bootstrapping with 1,000 simulations was used to estimate the average causal mediation effect (ACME), total effect, and proportion mediated. Mediators with ACME *p*-values < 0.05 and 95% CI not crossing zero were considered statistically significant. The analyses were conducted using standard R packages, including stats for regression models, pROC for ROC analysis, and mediation for mediation analysis.

## Results

A total of 467 patients hospitalized with pneumonia were included. Among them, 100 (21.4%) died within 30 days of admission. Baseline characteristics and comorbidities stratified by 30-day mortality are presented in Table 1. Compared with survivors, deceased patients were more likely to be female (28% *vs.* 49%, *p* < 0.001), present with confusion (75% *vs.* 13%, *p* < 0.001), and require higher levels of oxygen support (*p* < 0.001). The non-survivor group demonstrated significantly higher prevalence of multilobar infiltration, pleural effusion, hypertension, cardiovascular disease, cerebrovascular disease, dementia, and malignancy (all *p* < 0.01).

**Table 1:** Baseline characteristics and comorbidities of hospitalized patients with pneumonia, stratified by 30-day mortality status.

**Characteristic**	**Survived (*n* = 367)**	**Deceased (*n* = 100)**	***P***
Female sex, n (%)	180 (49%)	28 (28%)	<0.001
Confusion, n (%)	48 (13%)	75 (75%)	<0.001
Oxygen support level, n (%)			<0.001
Invasive	4 (1.1%)	11 (11%)	
Non-invasive	16 (4.4%)	11 (11%)	
Low-flow	347 (95%)	78 (78%)	
Multilobar infiltration, n (%)	173 (47%)	87 (87%)	<0.001
Pleural effusion, n (%)	82 (22%)	66 (66%)	<0.001
Hypertension, n (%)	212 (58%)	77 (77%)	<0.001
Diabetes mellitus, n (%)	162 (44%)	57 (57%)	0.022
Cardiovascular disease, n (%)	89 (24%)	46 (46%)	<0.001
Cerebrovascular disease, n (%)	49 (13%)	29 (29%)	0.002
Dementia, n (%)	37 (10%)	22 (22%)	0.008
Malignancy, n (%)	24 (6.5%)	23 (23%)	<0.001

**Notes.**

BP blood pressure n number % percentage

As shown in Table 2, patients who died had significantly older age, lower systolic blood pressure (SBP), and lower SpO_2_ on room air, as well as higher heart rate, temperature, and shock index (all *p* < 0.001). Deceased patients had significantly worse laboratory markers, including higher urea and lactate levels and lower albumin and lymphocyte counts (all *p* < 0.01). The HALP score was significantly lower among deceased patients compared with survivors (1.1 ± 1.4 *vs.* 2.5 ± 2.4, Δ = 1.4 (95% CI [1.0–1.8]), *p* < 0.001).

**Table 2:** Vital signs, laboratory parameters, and prognostic scores by 30-day mortality status.

**Variable**	**Survived (mean ± SD)**	**Deceased (mean ± SD)**	**Δ Mean (95% CI)**	***P***
Age, years	67.4 ± 16.9	75.7 ± 10.0	–8.3 (–10.9 to –5.7)	<0.001
Systolic BP, mmHg	120.5 ± 18.6	104.3 ± 23.9	16.2 (11.1 to 21.3)	<0.001
Heart rate, bpm	91.2 ± 14.3	107.6 ± 21.5	–16.4 (–20.9 to –11.9)	<0.001
Temperature, ^∘^C	37.0 ± 0.7	37.5 ± 0.9	–0.5 (–0.7 to –0.3)	<0.001
Shock index	0.78 ± 0.2	1.11 ± 0.4	–0.33 (–0.41 to –0.25)	<0.001
SpO_2_ (room air), %	84.3 ± 6.1	74.4 ± 10.4	9.9 (7.7 to 12.1)	<0.001
Lymphocyte count, 10^3^ /µL	1.49 ± 1.0	0.82 ± 0.9	0.67 (0.47 to 0.87)	<0.001
Urea, mg/dL	59.2 ± 38.6	93.0 ± 50.9	–33.8 (–44.6 to –22.9)	<0.001
Albumin, g/L	30.9 ± 11.7	22.2 ± 6.4	8.8 (7.0 to 10.5)	<0.001
Lactate, mmol/L	2.5 ± 5.0	4.1 ± 4.5	–1.7 (–2.7 to –0.6)	0.0019
HALP score	2.5 ± 2.4	1.1 ± 1.4	1.4 (1.0 to 1.8)	<0.001
CALLY index	4,420.9 ± 6,704.0	6,889.6 ± 8,130.2	–2468.7 (–4218.9 to –718.5)	0.006

**Notes.**

BP blood pressure SpO_2_ peripheral oxygen saturation bpm beats per minute CI confidence interval HALP hemoglobin, albumin, lymphocyte, and platelet score CALLY C-reactive protein × age/(lymphocyte × albumin) SD standard deviation

**Figure 1:**
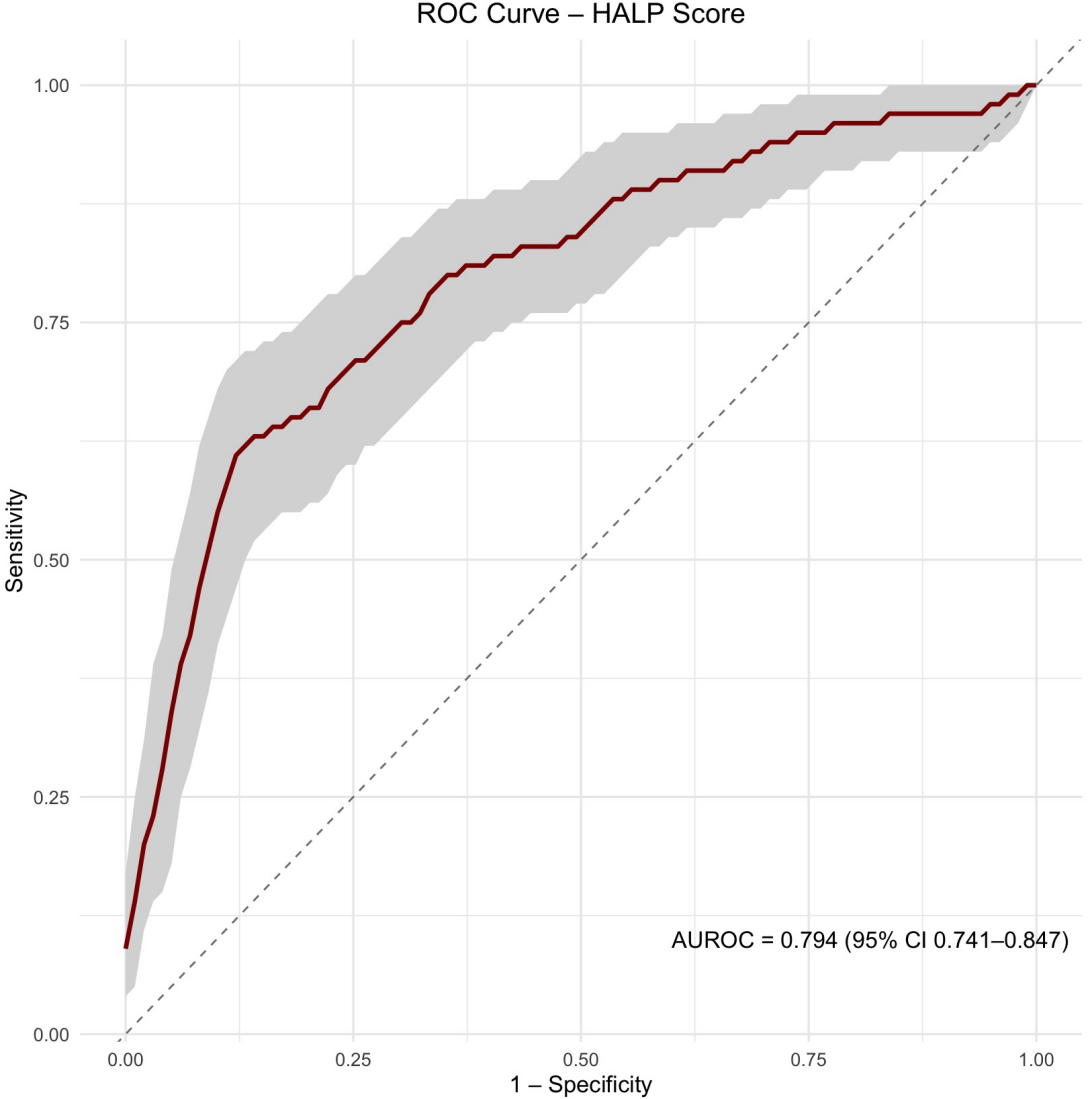
Receiver Operating Characteristic (ROC) curve of the HALP score for predicting 30-day mortality.

In ROC analysis (Fig. 1, Table 3), the HALP score showed moderate discriminative performance for 30-day mortality with an AUROC of 0.794 (95% CI [0.741–0.847]). At the optimal cutoff of 0.79, sensitivity was 62% (95% CI [52–72]%) and specificity was 88% (95% CI [84–91]%), corresponding to a positive likelihood ratio of 5.17.

**Table 3:** Diagnostic performance of the HALP score for predicting 30-day mortality.

**Score**	**AUROC (95% CI)**	**Threshold**	**Sensitivity (95% CI)**	**Specificity (95% CI)**	**+LR**	**–LR**
HALP	0.794 (0.741–0.847)	0.79	0.62 (0.52–0.72)	0.88 (0.84–0.91)	5.17	0.43

**Notes.**

AUROC Area under the receiver operating characteristic curve CI Confidence interval +LR Positive likelihood ratio –LR Negative likelihood ratio

**Figure 2:**
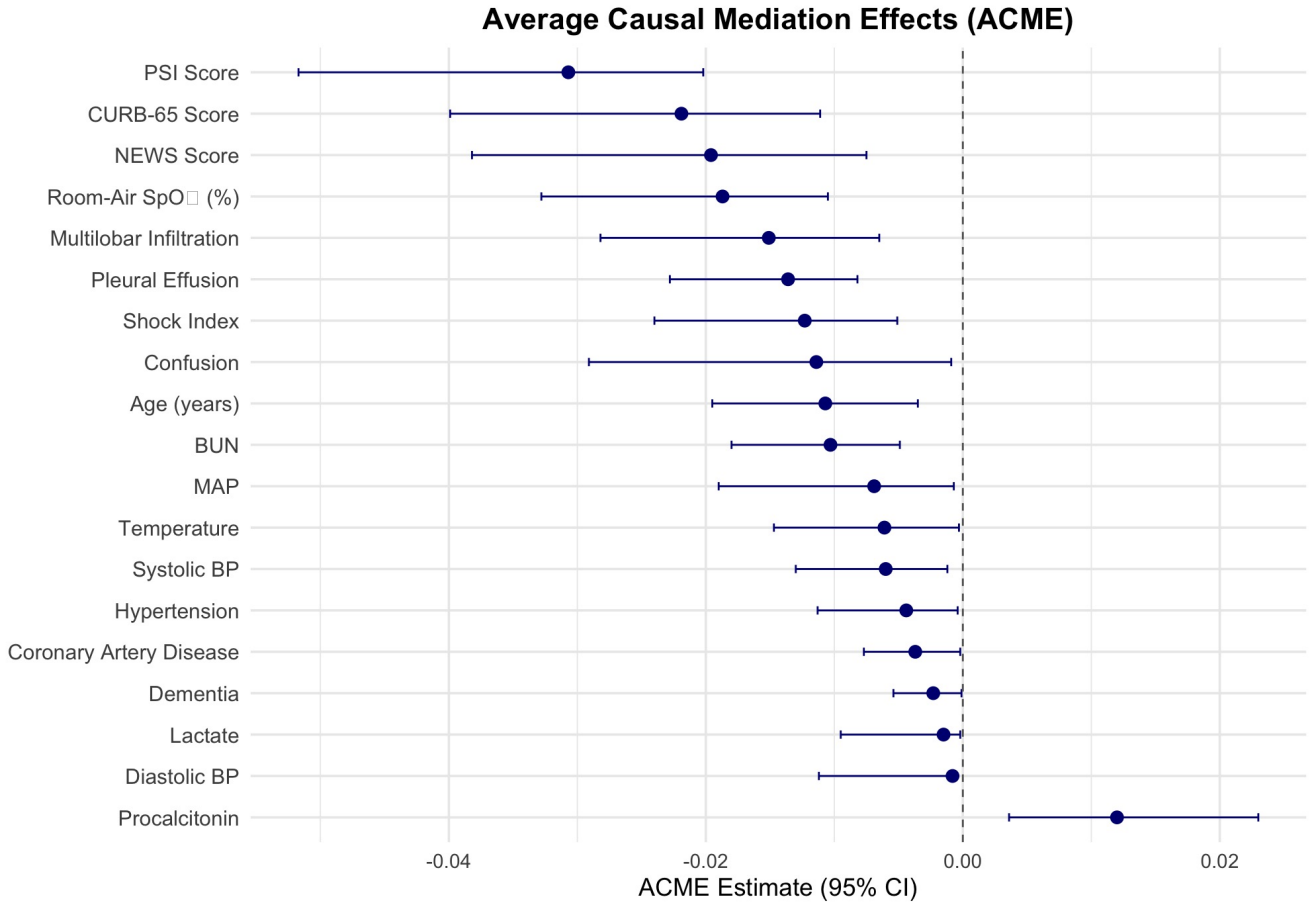
Causal mediation analysis of the association between HALP score and 30-day mortality, by significant mediators.

To investigate potential pathways linking the HALP score with 30-day mortality, an exploratory mediation analysis was conducted using 35 clinical variables as candidate mediators (Table 4, Fig. 2). In this analysis, negative ACME estimates indicate that lower HALP values are associated with increased mortality through the corresponding mediator. Of these, 20 demonstrated statistically significant average causal mediation effects (ACME, *p* < 0.05). The strongest mediators were PSI score (ACME = −0.0307, 95% CI [−0.0517 to −0.0202]), NEWS score (23.1% of the total effect), and CURB-65 score (22.5%). Room-air SpO_2_, multilobar infiltration, pleural effusion, and shock index also mediated ≥10% of the effect. Although factors such as coronary artery disease (*p* = 0.046), dementia (*p* = 0.030), lactate (*p* = 0.026), and diastolic blood pressure (*p* = 0.002) showed statistically significant ACME values, each accounted for <2% of the total effect. In contrast, variables such as neutrophil count (*p* = 0.532), creatinine (*p* = 0.526), and glucose (*p* = 0.800) did not show evidence of meaningful mediation.

**Table 4:** Causal mediation analysis of the association between HALP score and 30-day mortality, by significant mediators.

**Mediator**	**ACME estimate (95% CI)**	**Total effect (95% CI)**	**Proportion mediated (%)**	***P***
PSI Score	–0.0307 (–0.0517 to –0.0202)	–0.0925 (–0.1947 to –0.0484)	33.2	<0.001
CURB-65 Score	–0.0219 (–0.0399 to –0.0111)	–0.0973 (–0.2076 to –0.0564)	22.5	<0.001
NEWS Score	–0.0196 (–0.0382 to –0.0075)	–0.0850 (–0.1844 to –0.0448)	23.1	<0.001
Room-Air SpO_2_ (%)	–0.0187 (–0.0328 to –0.0105)	–0.1264 (–0.2521 to –0.0576)	14.8	<0.001
Multilobar Infiltration	–0.0151 (–0.0282 to –0.0065)	–0.1555 (–0.3014 to –0.0705)	9.7	<0.001
Pleural Effusion	–0.0136 (–0.0228 to –0.0082)	–0.1520 (–0.2884 to –0.0685)	8.9	<0.001
Shock Index	–0.0123 (–0.0240 to –0.0051)	–0.1111 (–0.2411 to –0.0471)	11.0	<0.001
Confusion	–0.0114 (–0.0291 to –0.0009)	–0.1033 (–0.2424 to –0.0498)	11.0	0.028
Age (years)	–0.0107 (–0.0195 to –0.0035)	–0.1839 (–0.3295 to –0.0863)	5.8	<0.001
Urea	–0.0103 (–0.0180 to –0.0049)	–0.1616 (–0.3060 to –0.0755)	6.4	<0.001
Mean Arterial Pressure	–0.0069 (–0.0190 to –0.0007)	–0.1292 (–0.2637 to –0.0562)	5.3	0.030
Fever	–0.0061 (–0.0147 to –0.0003)	–0.1738 (–0.3193 to –0.0894)	3.5	0.040
Systolic BP	–0.0060 (–0.0130 to –0.0012)	–0.1446 (–0.2812 to –0.0619)	4.2	0.008
Hypertension	–0.0044 (–0.0113 to –0.0004)	–0.1862 (–0.3304 to –0.0905)	2.4	0.026
Coronary Artery Disease	–0.0037 (–0.0077 to –0.0002)	–0.1895 (–0.3424 to –0.0958)	2.0	0.046
Dementia	–0.0023 (–0.0054 to –0.0001)	–0.1936 (–0.3469 to –0.0900)	1.2	0.030
Lactate	–0.0015 (–0.0095 to –0.0002)	–0.1923 (–0.3489 to –0.0889)	0.8	0.026
Diastolic BP	–0.0008 (–0.0112 to –0.0006)	–0.1948 (–0.3332 to –0.0868)	0.4	0.002
PCT	0.0120 ( 0.0036 to 0.0230)	–0.2019 (–0.3448 to –0.0984)	–5.9	0.008

**Notes.**

ACME Average causal mediation effect CI confidence interval HALP hemoglobin–albumin–lymphocyte–platelet index SpO_2_ peripheral oxygen saturation PSI Pneumonia Severity Index NEWS National Early Warning Score CURB-65 Confusion, Urea, Respiratory rate, Blood pressure, Age ≥65 score BP blood pressure MAP mean arterial pressure PCT procalcitonin

ACME estimates represent the indirect association between HALP score and mortality through the specified mediator. Negative ACME values indicate that lower HALP scores are associated with higher mortality risk through the corresponding variable.

To further evaluate whether the HALP score retained prognostic significance beyond established severity indices, an additional multivariable logistic regression analysis was performed using an 80/20 train–test split. The model included HALP score, Pneumonia Severity Index (PSI), CURB-65, National Early Warning Score (NEWS), and age.

In the training cohort, the HALP score remained independently associated with 30-day mortality (OR 0.60, 95% CI [0.45–0.81], *p* = 0.001) after adjustment for PSI, CURB-65, NEWS, and age (Table 5). PSI (OR 1.03, 95% CI [1.01–1.04], *p* < 0.001) and NEWS (OR 1.35, 95% CI [1.15–1.57], *p* < 0.001) were also independently associated with mortality, whereas CURB-65 and age were not significant in the adjusted model.

**Table 5:** Multivariable logistic regression analysis for 30-day mortality.

**Variable**	**OR**	**95% CI**	***p* value**
HALP score	0.60	0.45–0.81	0.001
PSI	1.03	1.01–1.04	<0.001
CURB-65	1.56	0.83–2.91	0.167
NEWS	1.35	1.15–1.57	<0.001
Age	0.99	0.95–1.02	0.460

**Notes.**

Model performance (test cohort): AUROC 0.903 (95% CI [0.817–0.970]); sensitivity 75.0% (95% CI [50.9–91.3]); specificity 94.6% (95% CI [86.7–98.5]); accuracy 90.4% (95% CI [82.6–95.5]).

Calibration: Hosmer–Lemeshow *χ*^2^ = 7.25, *p* = 0.510.

Nagelkerke R^2^: 0.613.

Collinearity: all VIF values < 3.5.

The model showed good calibration (Hosmer–Lemeshow *p* = 0.510), acceptable explanatory performance (Nagelkerke *R*^2^ = 0.613), and no evidence of problematic multicollinearity (all VIF values < 3.5). In the independent test cohort, the model demonstrated good discrimination with an AUROC of 0.903 (95% CI [0.817–0.970]). At a probability threshold of 0.50, sensitivity was 75.0% (95% CI [50.9–91.3]) and specificity was 94.6% (95% CI [86.7–98.5]).

## Discussion

The present study evaluated the prognostic value of the HALP score in patients with pneumonia, focusing on its ability to predict 30-day mortality. To date, few studies have examined this relationship in pneumonia, and our findings contribute to this limited body of evidence. The HALP score demonstrated strong discriminatory performance, with significantly lower scores observed in non-survivors compared to survivors; notably, the median HALP score in the non-survivor group was less than half of that in the survivor group. ROC analysis identified an optimal cutoff of 0.79, which predicted mortality with 88% specificity and 62% sensitivity. Comprising four routine laboratory parameters—hemoglobin, albumin, lymphocyte count, and platelet count—the HALP score represents a practical and efficient tool for early risk stratification in emergency departments, particularly where time-sensitive decision-making is essential. These results align with previous studies reporting an association between low HALP scores and increased mortality in various critically ill populations, including geriatric, septic, and oncologic patients (Çolak et al., 2023; Gursoy et al., 2024; Li et al., 2025). These findings suggest that the HALP score may serve as a useful and accessible biomarker for early identification of high-risk pneumonia patients.

After establishing a strong association between the HALP score and mortality, we conducted an exploratory mediation analysis to investigate potential pathways underlying this relationship. This analysis quantifies the proportion of the score’s effect on mortality that is direct *versus* mediated through other clinical factors. The most significant mediators identified were pneumonia severity indices, including PSI, NEWS, and CURB-65. PSI was the strongest mediator, explaining 33% of the score’s effect on mortality. PSI is a comprehensive risk model encompassing age, comorbidities, vital signs, and laboratory values; thus, a low score is associated with a high PSI, which increases mortality risk (Zaki et al., 2023). NEWS and CURB-65 also showed an inverse relationship with the score; for example, CURB-65 components such as confusion, elevated urea, and low blood pressure were more common among patients with low scores. In the mediation analysis, NEWS and CURB-65 accounted for 23% and 22% of the association between the score and mortality, respectively. These findings indicate that much of the score’s effect on mortality is linked to pneumonia severity. In other words, a low score generally reflects more severe pneumonia and a higher risk of death.

Another notable finding was that vital parameters, including room air oxygen saturation (SpO_2_) and shock index, also played significant mediating roles. Patients with low scores were more likely to experience severe hypoxemia and hemodynamic instability. Indeed, lymphopenia and hypoalbuminemia, components of the score, are commonly observed in sepsis and severe pneumonia, conditions frequently associated with hypotension and a higher risk of septic shock. The literature consistently reports that low SpO_2_ on room air and signs of shock are strong predictors of early mortality in pneumonia (Oh & Lee, 2023). Garcia-Vidal et al. (2008) found that most pneumonia patients who died within 48 h exhibited altered mental status, multilobar infiltration, and septic shock at admission. In our study, confusion, multilobar involvement, and shock index were also associated with the score and explained approximately 10% of its effect on mortality. Clinically, this suggests that a low score largely reflects patients presenting with decreased oxygen saturation and impaired perfusion, critical physiological disturbances that determine mortality. These findings suggest that low HALP scores are associated with clinical and physiological indicators of severe disease, including impaired oxygenation and hemodynamic instability.

Scoring systems such as PSI and CURB-65 are well-established tools in pneumonia management (Kaya et al., 2025). In our cohort, both scores were significantly elevated in non-survivors and showed strong inverse correlations with the HALP score. Consequently, comparing the prognostic performance of HALP to these traditional indices is pertinent. Our ROC analysis revealed an AUROC value of 0.79 for the HALP score. A recent large-scale cohort study involving 4,350 patients reported AUROCs of 0.83 and 0.73 for PSI and CURB-65, respectively, in predicting 30-day mortality. Scores such as qSOFA and SIRS demonstrated lower predictive accuracy with AUROCs below 0.60 (Tuta-Quintero et al., 2024). These findings suggest that HALP has moderate discriminative performance, although its standalone predictive accuracy appears lower than that of established severity indices such as PSI, CURB-65, and NEWS. However, its calculation relies on routinely available laboratory parameters, and may provide complementary biological information alongside established clinical scores. Importantly, HALP’s calculation relies on four routinely measured laboratory parameters which are available for nearly all patients. In contrast, PSI and CURB-65 require clinical and laboratory data that may be incomplete or unavailable, limiting their applicability in certain scenarios. Therefore, HALP may serve as a practical adjunct to existing scoring systems, especially when rapid risk stratification is needed. Moreover, by incorporating nutritional and inflammatory markers, the HALP score potentially captures pathophysiological dimensions of mortality risk not fully addressed by conventional clinical scores. Importantly, the HALP score should not be interpreted as a replacement for established pneumonia severity indices such as PSI or CURB-65. These tools remain the primary decision-support systems recommended in clinical guidelines for risk stratification and treatment setting decisions. Instead, the HALP score may provide complementary information reflecting the patient’s underlying immunological and nutritional status at admission. Because HALP is derived entirely from routinely available laboratory parameters, it may offer additional biological context alongside traditional clinical scores, particularly in early risk assessment in the emergency department.

Among the clinical and radiological findings of pneumonia, those most closely associated with mortality risk can be summarized as extensive lung involvement, development of complications, and the need for intensive care. The relatively high mortality rate in this cohort likely reflects a more severe hospitalized population, which may limit direct comparability with prior studies evaluating these scores in broader or lower-risk populations. In our study, 87% of deceased patients exhibited widespread infiltration involving multiple lobes. Multilobar involvement is an important indicator of pneumonia severity and is recognized as one of the minor criteria for severe pneumonia in clinical guidelines (Mandell et al., 2007). Involvement of multiple lobes indicates restricted lung capacity, impaired oxygenation, and possibly either high pathogen virulence or insufficient host defense. Therefore, mortality rates are significantly higher in multilobar pneumonia. Similarly, in our study, pleural effusion was nearly three times more common in the non-survivor group. The development of parapneumonic effusion or empyema complicates the clinical course of pneumonia and may necessitate invasive procedures such as drainage. Pleural effusion is also included as a risk factor in the PSI score and has been associated with poorer survival outcomes (Wei et al., 2021). In conclusion, multilobar involvement and the presence of pleural effusion should be considered red flags in patients with pneumonia, warranting close monitoring—preferably under intensive care conditions when feasible. Our study revealed that these findings were significantly more common among patients with low HALP scores and contributed to increased mortality. This underscores the need for clinicians to remain vigilant for these critical indicators, particularly in high-risk patients.

From a clinical perspective, the HALP score is not intended to replace established severity indices such as PSI, CURB-65, or NEWS. Instead, it may serve as a complementary biomarker reflecting the patient’s immunological and nutritional status at admission. Because the HALP score is derived from routinely available laboratory parameters, it may provide additional biological context during early risk assessment in emergency or acute care settings when interpreted alongside established clinical scoring systems. Importantly, although the HALP score showed lower standalone discriminative performance than PSI and NEWS, the additional multivariable analysis demonstrated that HALP remained independently associated with 30-day mortality after adjustment for established severity indices. This finding suggests that HALP may capture prognostic information not fully represented by conventional clinical scores alone.

In the management of pneumonia, laboratory tests play a role not only in supporting the diagnosis but also in predicting prognosis. In our study, several laboratory parameters showed significant differences in patients with fatal outcomes. Notably, albumin, lymphocyte count, urea, and lactate levels were markedly worse in the non-survivor group. Hypoalbuminemia is recognized as a marker of malnutrition and inflammation in critically ill patients and is a well-established indicator of poor prognosis in pneumonia (Liu et al., 2023). In our study, the mean albumin level among non-survivors was 22 g/L, indicating marked hypoalbuminemia, whereas survivors had a mean level around 31 g/L. Low albumin levels may impair immune function and increase tissue edema, thereby worsening the clinical course of pneumonia. Elevated urea levels, reflecting impaired renal function, are an important component of pneumonia risk scores. Similarly, lactate is considered a marker of tissue hypoperfusion (Huang et al., 2023). In pneumonia cases resulting in mortality, urea levels have been observed to be significantly higher, often indicating the presence of prerenal azotemia and renal hypoperfusion. Additionally, lactate levels were significantly elevated in non-survivors compared to survivors, with high lactate concentrations strongly associated with mortality in pneumonia patients. These findings support the development of shock and multiple organ failure in fatal cases of pneumonia.

### Limitations

This study has several important limitations. First, its single-center, retrospective design increases the risk of selection bias and makes the data accuracy dependent on the quality of patient records. Only patients with complete data required for score calculation were included in the final analysis, and no imputation was performed. Second, the study evaluated only 30-day mortality; longer-term outcomes such as survival, quality of life, and readmission rates were not assessed. Third, the prognostic performance of the HALP score identified in this cohort may be specific to the characteristics of the included patient population and may not generalize to different geographic regions or pneumonia subtypes (*e.g.*, community-acquired *vs.* hospital-acquired). Additionally, due to the retrospective observational design, causal inferences cannot be established, and the findings should be interpreted as associations rather than evidence of causality. Furthermore, while the causal mediation analysis provided valuable mechanistic insights, it relies on strong assumptions and has inherent limitations in inferring causality from observational data. Variables not included in the analysis—such as pathogen virulence, treatment quality, and patients’ functional status—may have influenced the HALP–mortality relationship. Also, laboratory values used to calculate the HALP score were measured only at admission, and potential changes in HALP over time were not evaluated. In addition, mediation analysis in observational data relies on several assumptions, including the absence of unmeasured confounding between the exposure, mediators, and outcome. Because the present study was retrospective, these assumptions cannot be fully verified, and the mediation findings should therefore be interpreted as exploratory associations rather than definitive causal pathways. In addition, time-to-event data were not consistently available, precluding survival analyses such as Kaplan–Meier or Cox regression. Lastly, laboratory measurements were obtained only at admission, and the prognostic value of temporal changes or serial assessments of the HALP score was not explored. Despite these limitations, the study makes a significant contribution to the literature by comprehensively evaluating the prognostic value of the HALP score. Future prospective, large-scale studies across diverse populations are warranted to better define the clinical utility of HALP and to guide targeted therapeutic strategies for patients with low HALP values.

## Conclusions

This study showed that the HALP score was independently associated with 30-day mortality in patients evaluated for pneumonia in the emergency department. The HALP score appears to reflect clinical severity and may provide complementary biological information alongside established severity scores. However, these findings represent observational associations and should be validated in prospective studies before clinical application.

##  Supplemental Information

10.7717/peerj.21442/supp-1 Supplemental Information 1 Raw dataset

## Additional Information and Declarations

### Data Availability

The following information was supplied regarding data availability: The dataset is available in the Supplementary File.

## References

[ref-1] American Thoracic Society, Infectious Diseases Society of America (2005). Guidelines for the management of adults with hospital-acquired, ventilator-associated, and healthcare-associated pneumonia. American Journal of Respiratory and Critical Care Medicine.

[ref-2] Chen J, Li Y, Zeng Y, Tian Y, Wen Y, Wang Z (2020). High mean platelet volume associates with in-hospital mortality in severe pneumonia patients. Mediators of Inflammation.

[ref-3] Çolak M, Uçkun S, Çoban H, Sarıoğlu N, Erel F (2023). Can the HALP score predict survival in severe COVID-19 pneumonia?. Postępy Higieny i Medycyny Doświadczalnej.

[ref-4] Das N, Bairwa M, Kant R, Goyal B, Bahurup Y (2024). Prognostic accuracy of lactate and procalcitonin in addition to national early warning score in patients with suspected sepsis—a cross-sectional study in a tertiary care center. International Journal of Critical Illness and Injury Science.

[ref-5] Fine MJ, Auble TE, Yealy DM, Hanusa BH, Weissfeld LA, Singer DE, Coley CM, Marrie TJ, Kapoor WN (1997). A prediction rule to identify low-risk patients with community-acquired pneumonia. The New England Journal of Medicine.

[ref-6] Frenzen FS, Kutschan U, Meiswinkel N, Schulte-Hubbert B, Ewig S, Kolditz M (2018). Admission lactate predicts poor prognosis independently of the CRB/CURB-65 scores in community-acquired pneumonia. Clinical Microbiology and Infection.

[ref-7] Garcia-Vidal C, Fernández-Sabé N, Carratalà J, Díaz V, Verdaguer R, Dorca J, Manresa F, Gudiol F (2008). Early mortality in patients with community-acquired pneumonia: causes and risk factors. The European Respiratory Journal.

[ref-8] GBD 2016 Causes of Death Collaborators (2017). Global, regional, and national age-sex specific mortality for 264 causes of death, 1980-2016: a systematic analysis for the Global Burden of Disease Study 2016. Lancet.

[ref-9] Gursoy V, Sadri S, Kucukelyas HD, Hunutlu FC, Pinar IE, Yegen ZS, Alkış N, Ersal T, Ali R, Ozkocaman V, Ozkalemkas F (2024). HALP score as a novel prognostic factor for patients with myelodysplastic syndromes. Scientific Reports.

[ref-10] Huang D, He D, Yao R, Wang W, He Q, Wu Z, Shi Y, Liang Z (2023). Association of admission lactate with mortality in adult patients with severe community-acquired pneumonia. The American Journal of Emergency Medicine.

[ref-11] Kaya M, Oduncuoğlu MK, Yıldırım H, Çoşkun A, Halici A, Tunc Y (2025). The effectiveness of CURB-65 and PSI scores in predicting hospital length of stay in patients diagnosed with community-acquired pneumonia in the emergency department. Van Medical Journal.

[ref-12] Kwok CS, Loke YK, Woo K, Myint PK (2013). Risk prediction models for mortality in community-acquired pneumonia: a systematic review. BioMed Research International.

[ref-13] Li H, Zhou Y, Zhang X, Yao R, Li N (2025). The relationship between hemoglobin, albumin, lymphocyte, and platelet (HALP) score and 28-day mortality in patients with sepsis: a retrospective analysis of the MIMIC-IV database. BMC Infectious Diseases.

[ref-14] Lim WS, Van der Eerden MM, Laing R, Boersma WG, Karalus N, Town GI, Lewis SA, Macfarlane JT (2003). Defining community acquired pneumonia severity on presentation to hospital: an international derivation and validation study. Thorax.

[ref-15] Liu Z, Wang Q, Wang H, Li J, Yuan Y, Yi G-Z (2023). Biomarkers for lipid and albumin metabolism in hospitalized patients with underlying diseases and community-acquired pneumonia caused by bacterial or SARS-CoV-2 infection. Journal of Inflammation Research.

[ref-16] Mandell LA, Wunderink RG, Anzueto A, Bartlett JG, Campbell GD, Dean NC, Dowell SF, File TM, Musher DM, Niederman MS, Torres A, Whitney CG, Infectious Diseases Society of America, American Thoracic Society (2007). Infectious Diseases Society of America/American Thoracic Society consensus guidelines on the management of community-acquired pneumonia in adults. Clinical Infectious Diseases.

[ref-17] Oh S, Lee K (2023). The new combination of oxygen saturation with age shock index predicts the outcome of COVID-19 pneumonia. SAGE Open Medicine.

[ref-18] R Core Team (2024). R: a language and environment for statistical computing. https://www.r-project.org.

[ref-19] Tuta-Quintero E, Bastidas AR, Guerrón-Gómez G, Perna-Reyes I, Torres D, Garcia L, Villanueva J, Acuña C, Mikler E, Arcila J, Chavez N, Riviera A, Maldonado V, Galindo M, Fernández M, Schloss C, Reyes LF (2024). Performance of risk scores in predicting mortality at 3 6, and 12 months in patients diagnosed with community-acquired pneumonia. BMC Pulmonary Medicine.

[ref-20] Viasus D, Nonell L, Restrepo C, Figueroa F, Donado-Mazarrón C, Carratalà J (2023). A systematic review of gene expression studies in critically ill patients with sepsis and community-acquired pneumonia. Biomedicines.

[ref-21] Wei X-S, Wang X, Ye L-L, Niu Y-R, Peng W-B, Wang Z-H, Zhang J-C, Zhou Q (2021). Pleural effusion as an indicator for the poor prognosis of COVID-19 patients. International Journal of Clinical Practice.

[ref-22] Zaki HA, Hamdi Alkahlout B, Shaban E, Mohamed EH, Basharat K, Elsayed WAE, Azad A (2023). The battle of the pneumonia predictors: a comprehensive meta-analysis comparing the Pneumonia Severity Index (PSI) and the CURB-65 score in predicting mortality and the need for ICU support. Cureus.

[ref-23] Zhai B, Chen J, Wu J, Yang L, Guo X, Shao J, Xu H, Shen A (2021). Predictive value of the hemoglobin, albumin, lymphocyte, and platelet (HALP) score and lymphocyte-to-monocyte ratio (LMR) in patients with non-small cell lung cancer after radical lung cancer surgery. Annals of Translational Medicine.

